# Habitat connectivity and in-stream vegetation control temporal variability of benthic invertebrate communities

**DOI:** 10.1038/s41598-017-00550-9

**Published:** 2017-05-03

**Authors:** K.-L. Huttunen, H. Mykrä, J. Oksanen, A. Astorga, R. Paavola, T. Muotka

**Affiliations:** 1 Department of Ecology & Genetics, University of Oulu, P.O.Box 8000, Oulu, 90014 Finland; 2 Freshwater Centre, Finnish Environment Institute, P.O.Box 413, Oulu, 90014 Finland; 3 Centro de Investigación en Ecosistemas de la Patagonia, Av. Almirante Simpson 471, Coyhaique, Chile; 4 Institute of Ecology and Biodiversity, Facultad de Ciencias Universidad de Chile, Las Palmeras 3425 Ñuñoa - Casilla 653, Santiago, Chile; 5 Oulanka Research Station, University of Oulu Infrastructure Platform, Liikasenvaarantie 134, Kuusamo, 93900 Finland; 6 Natural Environment Centre, Finnish Environment Institute, P.O.Box 413, Oulu, 90014 Finland

## Abstract

One of the key challenges to understanding patterns of β diversity is to disentangle deterministic patterns from stochastic ones. Stochastic processes may mask the influence of deterministic factors on community dynamics, hindering identification of the mechanisms causing variation in community composition. We studied temporal β diversity (among-year dissimilarity) of macroinvertebrate communities in near-pristine boreal streams across 14 years. To assess whether the observed β diversity deviates from that expected by chance, and to identify processes (deterministic vs. stochastic) through which different explanatory factors affect community variability, we used a null model approach. We observed that at the majority of sites temporal β diversity was low indicating high community stability. When stochastic variation was unaccounted for, connectivity was the only variable explaining temporal β diversity, with weakly connected sites exhibiting higher community variability through time. After accounting for stochastic effects, connectivity lost importance, suggesting that it was related to temporal β diversity via random colonization processes. Instead, β diversity was best explained by in-stream vegetation, community variability decreasing with increasing bryophyte cover. These results highlight the potential of stochastic factors to dampen the influence of deterministic processes, affecting our ability to understand and predict changes in biological communities through time.

## Introduction

Composition of biological communities at any given site or time is an outcome of deterministic and stochastic processes. Deterministic perspectives, such as the species-sorting concept^1^, view communities as a result of interactions between species and their biotic and abiotic environment, with each species having its own predictable niche. Due to environmental differences among habitats, each locality is advantageous for some species while disadvantageous for some others. At the other extreme, stochastic perspectives highlight random colonization and extinction and ecological drift, i.e. random population fluctuations, as mechanisms leading to communities where, in the strictest sense, all species have equal requirements and probability of colonizing a site^2, 3^. This may lead to high β diversity (i.e. high dissimilarity) in community composition among sites and times, potentially reinforced by priority effects - the impact of a particular species on community assembly due to prior arrival at a site^4^. Here we define ‘stochastic process’ as any process that gives rise to patterns of species diversity, relative abundance and community composition indistinguishable from chance. By contrast, any process creating structure that cannot be obtained by chance and whose effect is dependent on species identity is considered ‘deterministic’^5^.

Spatial β diversity is typically related to dispersal limitation or environmental heterogeneity and productivity^6–8^. Although several previous studies have assessed site-specific changes in community composition through time^9–11^, they have not focused specifically on temporal β diversity. Indeed, temporal β diversity has been much less studied than its spatial counterpart, yet partly the same mechanisms should regulate both aspects of β diversity^12^. Variable environments tend to support high temporal community variability by favoring different species at different times^13, 14^. Alternatively, very high environmental variability (e.g. high disturbance frequency) may act as a strong environmental filter whereby only the most tolerant taxa persist in frequently changing conditions, resulting in a negative relationship between temporal β diversity and environmental variability^15, 16^. It has been reported, for example, that stream invertebrate communities vary less through time at sites with highly variable flow conditions^17^. Spatial and temporal variability may also interact if spatially heterogeneous habitats provide refugia during adverse conditions, thereby dampening the effects of environmental variability^18, 19^. Similarly, abundant vegetation reduces temporal variability of stream invertebrate communities^11^, likely via the same mechanism as within-site habitat heterogeneity.

Stochastic processes may mask the influence of deterministic factors on community dynamics, potentially obscuring the mechanisms of community variability across space and time^12^. Sampling effects are an important source of stochasticity in community patterns. We define sampling effect to arise when there is random recruitment of individuals into local communities from the regional species pool^12^. Temporal turnover depends on the number of species at any particular year (temporal α-diversity), causing uncertainty as to the mechanism of observed community variability; that is, is it caused simply by random differences in α-diversity among samples^20^. Sampling effects may also arise from differences in total number of species across the study period (temporal γ-diversity)^12, 21^. Higher β diversity may then be expected at sites of high compared to low γ-diversity because, with an increasing number of species, the probability of observing some species only once or twice also increases.

In addition to sampling effects, community variability may arise from stochastic ecological processes such as chance colonization, random changes in local species abundances and priority effects. For example, communities in isolated sites may exhibit more stochastic variability than those in better connected sites^22^, as high connectivity increases the influx of immigrants. Spatial location can be particularly important in streams that form dendritic networks enabling continuous dispersal and re-colonization through downstream drift and adult flight along (and across) river corridors^23^. The upmost headwaters are often weakly connected to other parts of the river network, and headwater communities may therefore exhibit higher temporal turnover than those in mid-order reaches.

We examined the temporal (inter-annual) β diversity of benthic invertebrate communities in 23 near-pristine streams in northern Finland across 14 consecutive years. This was done separately for each stream, with the mean community dissimilarity for consecutive year pairs being our measure of site-specific β-diversity. Correspondingly, the mean annual richness across the 14 years was our estimate of α-diversity, and the total species number across all study years provided the temporal γ-diversity for a site. Our aim was to examine (*i*) if, and by how much, the observed level of temporal β diversity differed from that expected by chance; and (*ii*) to explore if the key environmental drivers suggested to determine spatial β diversity also explain temporal β diversity, even after controlling for stochasticity, indicating that these factors operate through mainly deterministic pathways. We predicted that (*i*) increased habitat connectivity should decrease temporal β diversity, with a higher amount of propagules promoting community stability but, if dispersal among sites is neutral, the importance of connectivity should diminish when stochastic variation is accounted for. We expected (*ii*) a positive relationship between temporal γ-diversity and temporal β diversity if the pattern is caused by sampling effects. We also expected that (*iii*) flow-related extreme events pose a strong disturbance filter, allowing only the most tolerant taxa to persist at a site; therefore, temporal β diversity should decrease with increasing disturbance. Finally, we expected (*iv*) within-site habitat heterogeneity and in-stream vegetation to dampen temporal variability of invertebrate communities by affording more refugia for the invertebrates during adverse flow conditions.

## Results

**Figure 1:**
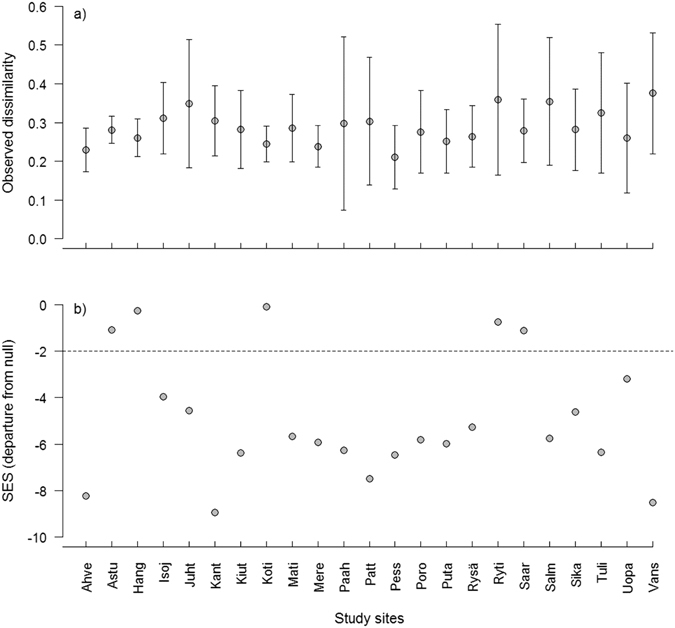
Site-specific temporal β diversity. The mean (±1 SD) among-year dissimilarity, calculated for consecutive year pairs, for each study site expressed as (**a**) observed dissimilarity (Bray-Curtis index) and as (**b**) departure from null expectation. The dashed line represents the limit below which a community is interpreted as being more stable than expected by chance (β_dep_ < −2). Above the line, variation in community composition does not differ from random expectation.

Our data contained overall 129 macroinvertebrate taxa. Mean taxa richness at a site per year (temporal α-diversity) was 31, ranging from 25 to 40. Site-specific total amount of taxa across all study years (temporal γ-diversity) was on average 64, ranging from 50 to 81. The mean among-year Bray-Curtis dissimilarity, i.e. observed temporal β diversity, ranged from 0.210 to 0.375 (mean: 0.288; Fig. 1a), whereas β diversity compared to that expected by chance (β_dep_) varied from -0.085 to -8.948. A great majority of sites (18 out of 23) had lower temporal β diversity (i.e. higher stability) than expected by chance (β_dep_ < −2; Fig. 1b).

**Figure 2:**
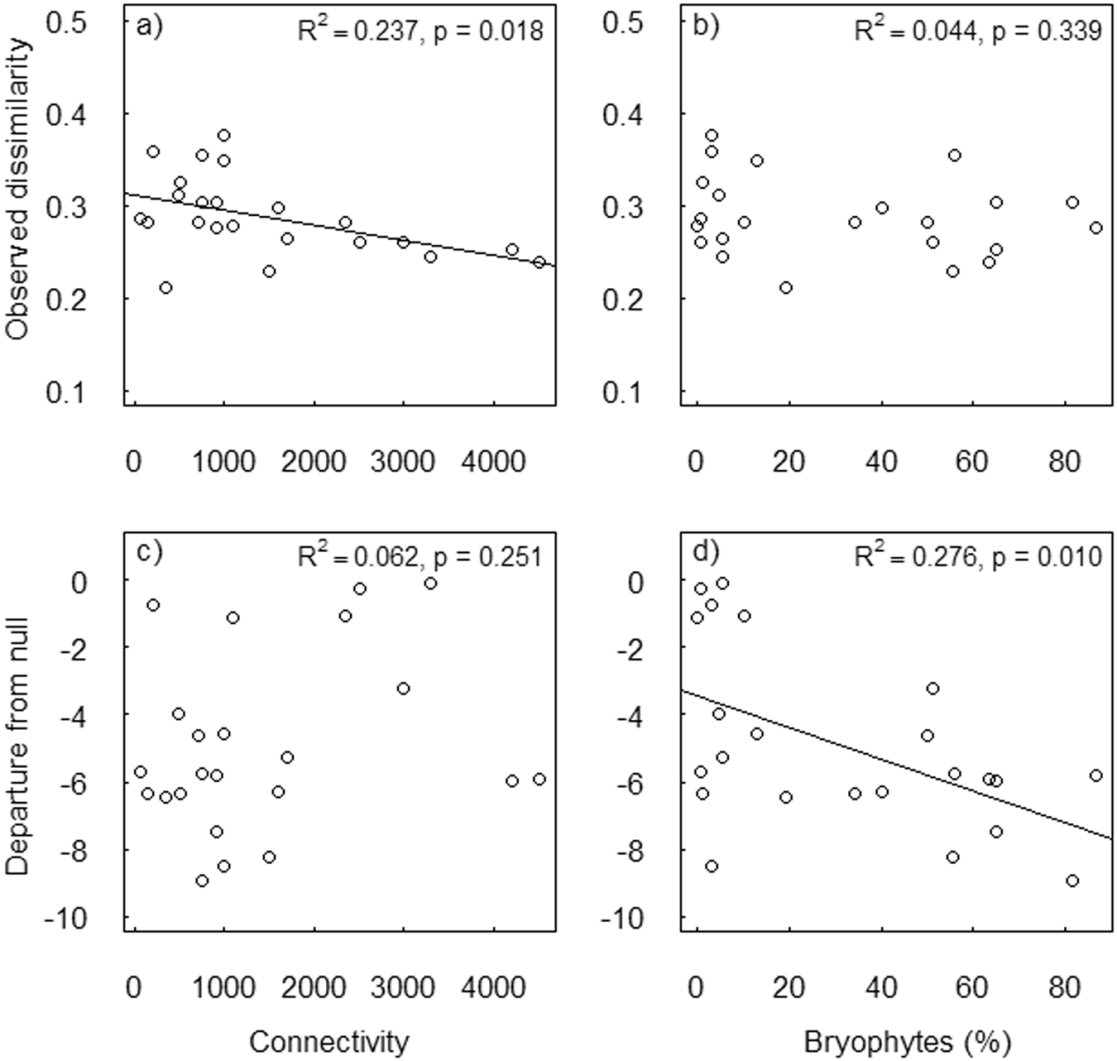
Regressions between temporal β diversity and selected environmental variables. Univariate linear regressions between the observed dissimilarity (top row: a&b) or deviation from the null expectation (bottom row: c&d) and connectivity (i.e. riffle area within a 500-m buffer of a study reach, m^2^) and bryophyte cover.

Connectivity was the only variable included in the best model for the observed temporal β diversity, with weakly connected sites exhibiting more variation in community composition across years. The importance value for connectivity approached the threshold of 0.80, whereas all other variables obtained very low importance values, emphasizing the primary role of connectivity in explaining temporal β diversity of invertebrate communities (Table 1; see also Fig. 2a).

**Table 1:** Standardized regression coefficients for the best models (Δ_AICc_ < 2) explaining temporal β diversity in stream macroinvertebrate community composition based on Bray-Curtis dissimilarity values (average dissimilarities between consecutive years) on log(x + 1)-transformed data.

a) Dependent: Bray-Curtis dissimilarity, observed (β_obs_)								
Importance	BMI	Bryophytes	Simpson	Temp.	Connectivity	Gamma	adj.R²	Δ_AICc_
	x	x	x	x	−0.487	x	0.201	0
	0.178	0.213	0.178	0.227	**0.777**	0.185		
b) Dependent: Bray-Curtis dissimilarity, departure from null (β_dep_)								
Importance	BMI	Bryophytes	Simpson	Temp.	Connectivity	Gamma	adj.R²	Δ_AICc_
	x	−0.606	x	x	0.379	x	0.354	0
	x	−0.525	x	x	x	x	0.241	1.870
	0.252	**0.901**	0.314	0.257	0.524	0.195		

Dependent variables are (a) observed dissimilarity (β_obs_) and (b) departure of the observed dissimilarity from the null expectation (β_dep_). x Denotes that a variable was not included in that model. The overall importance across all candidate models is also presented for each predictor; the highest importance value is given in bold. BMI = bed movement intensity, Temp = water temperature.

When stochastic variation was controlled for by using departure from null as response variable, both of the best models (Δ_AICc_ < 2) included bryophyte cover as the key variable (importance value: 0.90) (Table 1). The relationship between community dissimilarity and bryophyte cover was negative, that is, temporal β diversity decreased with increased amount of in-stream vegetation (Fig. 2b). Connectivity was also included in one of the best models. However, the importance value for connectivity was much lower (0.52) and thus clearly inferior compared to bryophyte cover.

## Discussion

We studied the temporal β diversity of stream macroinvertebrate communities with the aim of disentangling the relative importance of deterministic vs. stochastic factors to community variability. For the majority of sites, site-specific temporal β diversity across years was significantly less than expected by chance, indicating that macroinvertebrate communities in these near-pristine forest streams were temporally stable. Connectivity among suitable habitats within a stream was clearly the most influential determinant of the observed community variability. However, the negative relationship between connectivity and temporal β diversity disappeared once stochastic effects were accounted for, implying that connectivity contributed to community variability mainly via stochastic processes. Connectivity within stream networks likely relates to dispersal probability, with more connected sites receiving a higher influx of propagules, thus exhibiting a lower likelihood of stochastic extinctions and less temporal variability in community composition than more isolated sites^24^. This supports the view of dispersal as a stochastically-driven process that may induce variation in community composition not only among sites but through time as well^2, 3^. Stream metacommunity studies frequently use Euclidean or topographic distances between study sites as a surrogate for connectivity. While topographic distances to other streams may indeed be relevant for stream insects in arid landscapes where streams frequently dry out^25, 26^, all of our sites are perennial and do not dry completely at any time. Furthermore, local communities are connected to many other sources of colonization than the sites that are included in a given study, and the availability of the most likely recolonization sources may be a better surrogate for dispersal than distances to other sampling sites in (mainly) other streams^27, 28^.

After accounting for stochastic effects connectivity was no longer important. Temporal β-diversity of invertebrate communities was instead best explained by in-stream vegetation, with more stable communities at sites with abundant bryophytes, whereas other factors (e.g. disturbance, habitat heterogeneity) shown to be important drivers of spatial (and, less so, temporal) community variability were of marginal importance. The strong negative relationship between bryophyte abundance and temporal β-diversity corroborates our earlier finding about the key role of bryophytes to stream invertebrate community stability^11^. If bryophytes are very abundant, they may create a homogeneous stream habitat, which may then explain low β diversity. Nevertheless, it seems more likely that the negative relationship is related to bryophytes providing refugia against flow-related disturbances and thus increasing community stability through time. In addition, although only few invertebrates directly consume bryophytes, they afford abundant food resources by acting as substrate for epiphytic algae and by enhancing organic matter retention^29, 30^. The time span of our previous study was much shorter, however, only four years^11^. Recent work suggest that solution to the ‘neutral vs. niche’ debate may depend on the scale of the study, with stochastic factors becoming more important as the size of the sampling plot decreases^31, 32^. Our finding of deterministic control by bryophytes over temporal β-diversity of stream invertebrate communities is therefore substantiated by the fact that the same relationship seems to hold at differing temporal ‘windows of opportunity’, from inter-annual^11^ to decadal (this study). Similarly, other studies have reported a negative relationship between substrate heterogeneity and temporal turnover, likely reflecting partly the same mechanisms as bryophytes in our study^18, 19^. However, we did not detect such a stabilizing effect of substrate heterogeneity on inter-annual variation in community composition.

Habitat stability has been frequently evoked as a key factor promoting stability of biological communities^13, 33^. We measured habitat stability at a scale directly relevant to benthic organisms, using stone movement as our measure of disturbance^34, 35^. While practically all previous studies using the same technique to quantify in-stream disturbance have continued for a relatively short period, typically a few months, we monitored stone movement for five successive years, guaranteeing that our study encompassed major flow events from a drastic drought in 2006 to peak flows during spring floods^36^. Running water ecosystems are traditionally thought of as highly disturbance-prone environments where frequent and unpredictable disturbances exert strong control over community structure; streams have even been suggested to be one of the most disturbance-prone (‘flashiest’) ecosystems^37, 38^. In our study, however, community stability was unrelated to bed movement intensity. It has been argued that stones placed on substrate surface move more easily during high flows than do more embedded natural stones^39^. However, our estimates of stone movement were clearly lower than in most previous studies using the same technique to assess flow-related disturbance in streams. Therefore, if anything, our results should be too liberal, and bed movement intensity in these and other similar boreal streams may be even lower than observed in this study. While the influence of bed disturbance as a driver of the temporal β-diversity of invertebrate communities in these near-pristine streams seems negligible, we cannot exclude the possibility that this pattern may simply reflect the somewhat restricted disturbance gradient of our study: even the highest spring floods moved only about one-third of the stones, compared to 100% in similar studies^34, 35^. Nevertheless, disturbances that cause a complete streambed turnover must be very rare and the disturbance gradient detected in our study seems realistic for unmodified boreal streams.

One of the key challenges to ecologists trying to understand patterns of community variability, either through space or time, is to disentangle deterministic patterns from those induced by chance, especially those resulting from pure sampling effect. Null models have proved to be a valuable tool for controlling stochasticity, providing a measure for the deviation of the observed dissimilarity from null expectation^20, 21^. Although the use of null models to test if observed β diversity deviates from random expectation is an emerging practice in studies on spatial turnover^40–42^, they are still rarely used for assessing temporal β diversity of biological communities. Our results show that the identity and relative importance of explanatory variables may vary profoundly depending on whether community variability is measured as observed dissimilarity or departure from null. Such deviating outcomes indicate that stochastic processes may dampen, or even completely obscure, the influence of deterministic processes on β diversity, thus affecting our ability to understand and predict changes in biological communities through time. In our case, failing to consider stochasticity would have resulted in a complete neglect of the importance of local habitat filters, particularly in-stream vegetation. Obviously, reliable data on dispersal capacities of individual species would allow the construction of ecologically more realistic null models, and one could then expect stronger deterministic effects of connectivity on temporal variability of weak dispersers whereas strong dispersers would be less dependent on connectivity (for a spatial counterpart, see refs 43 and 44). Finally, from an applied perspective, the strong role of habitat connectivity to temporal β diversity suggests that the recovery of stream communities from human-induced disturbances may be constrained by dispersal limitation of lotic taxa^45^. Consequently, the success of restoration and conservation programs may be compromised by habitat fragmentation that causes discontinuity along the stream network, emphasizing the need for a landscape-scale approach to stream management and conservation planning.

## Methods

### Study sites

We conducted our study in 23 streams in the Finnish part of the Koutajoki drainage basin in northeastern Finland, just south of the Arctic Circle (66–67°N, 28–30°E). Koutajoki basin is geologically diverse with extensive deposits of calcareous rocks. Vegetation is highly variable, dominated by pine forests and peatlands. Our study sites are first-to-second order streams with catchments ranging from 2.3 to 50.1 km^2^ (mean 15.2 km^2^). Many of them are located within a nature conservation reserve, Oulanka National Park, which represents the westernmost remnants of pristine taiga forests^45^. All the streams drain mixed forests and bogs with minimal anthropogenic impact, less than ten percent of their catchments being modified by any land use activities (mainly forestry). Headwater streams of the region are highly oligotrophic with circumneutral to slightly alkaline waters with low humic content^46^. All our study streams have permanent flows, with discharge peaks tracking closely the spring snowmelt (late April to early May). Site characteristics are described in more detail in Table S1.

### Sampling of benthic invertebrates

We sampled benthic invertebrates in autumn, about four months after the spring flood. Sampling was conducted during 14 consecutive years (2000 to 2013), always by the same field crew. In each stream, we delineated a riffle section of about 100 m^2^ where all biological and environmental sampling (except connectivity) was conducted. We then collected a 2-min kick-net (mesh size 0.3 mm) sample, consisting of four 30-s subsamples which were subsequently pooled. Each sample aimed to cover different microhabitat types available in a riffle in relation to their proportional availability. Such a sample covers 1.3 m^2^ of the stream bed, capturing about 75% of all species present in a riffle, mainly missing species that occur only sporadically in streams^47^. Samples were preserved in 70% ethanol in the field and later processed in the laboratory. All individuals were sorted, counted and identified to the lowest feasible level, mainly species (65% of all individuals) or genus (27%), except for Diptera and some Limnephilidae caddis larvae which were identified to family level. Chironomids were not counted every year and were therefore excluded from all analyses. Benthic invertebrate fauna in our study area is strongly dominated by aquatic insect, with non-insects contributing generally less than 10% of all benthic invertebrates^46^.

### Explanatory variables

We quantified environmental stability as bed disturbance intensity and frequency by monitoring the movement of individual stones within a reach. We first quantified substrate size distribution at a site by measuring the longest dimension for at least 100 randomly selected particles. We then collected stones from the immediate surroundings of the study reach corresponding to the 50^th^, 75^th^ and 90^th^ percentiles of the substrate size distribution at a site. We marked each stone individually using waterproof paint. In late May 2005, the stones were arranged in 12 transects perpendicular to the flow, each transect consisting of three regularly spaced stones, one in each size class, in a random order. We then monitored stone movement twice a year until autumn 2009: after the spring flood in early June and concurrently with benthic sampling in September. On each occasion, we recorded if the stones had moved. Displaced stones were returned to their original position or, if disappeared, replaced with a similar-sized stone. We quantified bed movement intensity (BMI) as the mean percentage of stones moved (or turned) across the whole study period^34^. We emphasize that as we did not sample embedded particles or bed clusters, this index should be considered strictly as an index of bed disturbance^48^. We also calculated bed movement frequency (proportion of monitoring periods with more than 20% of stones moved) as well as maximum bed movement. However, as all three measures were strongly correlated (r = 0.87–0.95), we used only BMI in further analyses. Bed movement intensity differed between seasons: spring floods moved stones much more frequently than did the summertime flows (Fig. S1). Unfortunately, we were unable to monitor bed movement across all study years, but based on data from a meteorological station at Oulanka Research Station, located centrally in our study area, the five monitoring years included both an extreme drought year (once-in-a-50-year drought in 2006) and an exceptionally wet year (2009). We therefore consider the five sampling years to be well representative of the annual precipitation regime (and, consequently, stream discharges) during the whole study period. Furthermore, Spearman rank correlations showed that the ranking of sites in terms of disturbance intensity remained fairly constant during the study period (mean r_s_: 0.544), with only small rank changes between sites at intermediate positions along the disturbance gradient.

We measured in-stream habitat heterogeneity as substratum diversity (Simpson index, 1/D) based on particle size distribution in ten 50 × 50 cm quadrats (modified Wentworth scale from silt (0) to large boulder and bedrock (9)^36^. Percentage cover of bryophytes was estimated visually at twenty randomly placed 50 × 50 cm quadrats at each site. Replicate measurements were averaged to give a single value for each variable. While these variables may vary through time, our repeated measurements in the 23 streams included in this study across three years (2000 to 2002) suggest that the relative ranking of sites remains very constant across years (r_s_ = 0.831 for bryophytes, 0.856 for substrate size; H. Mykrä, unpublished data). We also measured stream water temperature at 30-min intervals from late May to early October for four consecutive years (2009–2012) at each site using data loggers (WT-HR 1000 mm, TruTrack Ltd, New Zealand). Daily averages were used to calculate mean water temperature for each site. The mean value across the four monitoring years was then used in data analysis. Although temperature variation across the whole study period was relatively minor, Spearman rank correlations showed that the site ranking in terms of water temperature across the four years of monitoring remained highly consistent (r_s_ = 0.911). Finally, connectivity was measured as the relative isolation of a site within a stream network by estimating, based on site visits, availability of riffle habitat (as m^2^) within a 500 m buffer in both upstream and downstream directions from the sample reach. As our streams are characterized by distinct riffle-pool sequences, the 1000-m distance surveyed for riffle availability in each stream included usually several riffle sections separated by slow-flowing, deep pools. While any buffer size is more or less arbitrary as it cannot be reliably related to (typically unknown) dispersal distances of stream invertebrates, we consider this measure reasonably sensitive in capturing most dispersal events along the stream network^49^. Occasionally (in 4 of the 23 streams) this distance also included other nearby tributaries (for a similar approach, see ref. 28). We measured connectivity also by calculating the number of adjoining tributaries within a 2-km buffer in both upstream and downstream direction of a study site. As the two measures of connectivity gave closely similar results, we used riffle area (length × stream width) per 1000 m river length) in all analysis as we consider it to better represent local-scale connectivity. Individual dispersal events can occasionally extend beyond the 500-m distance used by us^50, 51^. We assessed the role of such large-scale connectivity by measuring straight-line distances to five nearest streams (other riffles in the same stream not included). Although more complicated measures that take landscape topography into account^26, 43^ could have been used, we think that the simple Eulidean distance provides a sensible approximation of the relative isolation of a site within the landscape. In univariate regression, this measure of connectivity was unrelated to interannual dissimilarity (departure of the observed dissimilarity from null expectation, see below; R^2^ = 0.028) and was therefore not included in the modeling approach.

### Data analysis

#### Temporal variability of community composition

We analyzed temporal β diversity, i.e. inter-annual variability in community composition, by using Bray-Curtis dissimilarity index based on log(x + 1)-transformed abundance data; low index values represent low temporal β diversity and thus high community stability through time. We computed temporal β diversity (β_obs_) separately for each site by calculating Bray-Curtis dissimilarity first across all consecutive year pairs (13 pairs), and then used the mean dissimilarity across years for a site as response variable in subsequent analyses.

To assess whether the observed level of temporal turnover deviates from that expected by chance, we used a null model approach^12, 20, 42^ (function *nullmodel*, algorithm *swsh_both_r*) with vegan package version 2.2–0 in the R program (http://cran.r-project.org/)^52^. For each site, we constructed a quantitative null model that first randomly shuffles species presences so that species richness for each sample (α), species frequencies, and site-specific γ diversity are retained. Individuals are then distributed randomly over non-zero cells for each row so that sample totals are preserved. This procedure thus produces the mean (across 1000 iterations) expected temporal β diversity (β_exp_) for consecutive years across the study period for a null community with fixed γ-diversity, given annual variation in taxa richness and total abundance. To estimate the level of temporal β diversity independent of chance alone we then calculated departure of the observed dissimilarity from the null expectation, expressed as effect size (β_dep_ = (β_obs_ − β_exp_)/SD β_exp_). This value shows the number of standard deviations that the observed dissimilarity deviates from that expected by chance^21^. Negative β_dep_ values indicate that community composition among consecutive years is less dissimilar than expected by chance (low temporal β diversity), while positive values indicate more dissimilar communities than expected (high temporal β diversity).

Null models in community ecology are often criticized of having overly simplistic randomization structure, mainly because the kind of detailed information about, for example, species’ dispersal capacities required for more sophisticated null models is usually lacking^53^; therefore, null models that allow one to reliably disentangle ecological processes that structure communities “remain elusive”^54^. To this end, our null model is not ecologically realistic, but rather a general null in the sense of species equality^2^ in terms of dispersal ability. While a regional null model would have provided more robust patterns (more distinct departure from the null), distinguishing temporal β diversity from its spatial counterpart would not have been possible then.

Differences between the observed and expected dissimilarity may arise from temporal autocorrelation, with communities between consecutive years being more similar than expected by chance - a pattern inherent in real communities but lacking from the null model. To estimate the effect of temporal autocorrelation we used generalized additive mixed model with observed dissimilarity across all possible year pairs as response variable and smoothed time step as the explanatory variable^55^. The level of autocorrelation for the one-year time step was low (decrease of 0.03 in observed Bray-Curtis values), and practically equal among sites, indicating that any effect of temporal autocorrelation on our results is small.

#### Relating temporal turnover to environmental variables

We used multimodel inference^56, 57^ in multiple linear regressions to examine the relationships between environmental variables and temporal β diversity. Specifically, we were interested in the influence of environmental stability (BMI), habitat heterogeneity (Simpson diversity), in-stream vegetation, water temperature, temporal gamma diversity, and connectivity on community variability. To restrict the number of candidate models, only individual variables were explored (no interaction terms), the total number of candidate models being 64. Initially, we also included two measures of productivity (algal accrual rate, organic matter standing stock) and water chemistry (pH, total phosphorus, water color) in our analysis, but as these varied little among the study sites and were of negligible importance in regression models, we excluded them from all analyses to keep the models relatively simple (and interpretable) and to avoid multicollinearity. We compared the explanatory power of models using Akaike Information Criterion with small-sample correction (AIC_c_), the best model being the one with the lowest AIC_c_ score. Differences in AIC_c_ scores between each model and the best model (Δ_AICc_) express the loss in information if the best model is not used. Models with Δ_AICc_ < 2 are interpreted as having equal support. We used model weights to compare the relative importance of explanatory variables. Summing model weights across all models that include a certain variable gives the importance value for that variable, allowing a comparison among variables^56, 57^. To aid interpretation, we used a threshold importance value of 0.80 below which terms were regarded unimportant. This threshold generally yields good properties in terms of type I and type II error rate^58^. The R package MuMIn^59^ was used to relate community variability to environmental variables. To identify processes through which the explanatory factors affected temporal β diversity (deterministic vs. stochastic), both the observed level of β diversity (β_obs_) and departure from null (β_dep_) were used as response variables. If an explanatory variable affects temporal β diversity through stochastic processes, its role should become non-significant, whereas factors reflecting deterministic processes will remain (or become) significant, once stochastic effects are controlled for.

### Data availibility

Data available from the Dryad Digital Repository: http://dx.doi.org/10.5061/dryad.vt68r.

## Electronic supplementary material

Supplementary material
