# Supplementary material for: Habitat connectivity and in-stream vegetation control temporal variability of benthic invertebrate communities
Source: Sci Rep. 2017 May 3;7:1448. doi: 10.1038/s41598-017-00550-9 (PMC5431217; doi:10.1038/s41598-017-00550-9)

Habitat connectivity and in-stream vegetation control temporal variability of benthic invertebrate communities

K.-L. Huttunen[*1], H. Mykrä[2], J. Oksanen[1], A. Astorga[3,4], R. Paavola[5] and T. Muotka[1,6]

1 Department of Ecology & Genetics, University of Oulu, Finland

2 Freshwater Centre, Finnish Environment Institute, Finland

3 Centro de Investigación en Ecosistemas de la Patagonia, Chile

4 Institute of Ecology and Biodiversity, Facultad de Ciencias Universidad de Chile, Chile

5 Oulanka Research Station, University of Oulu Infrastructure Platform, Finland

6 Natural Environment Centre, Finnish Environment Institute, Finland

[*] Corresponding author: kaisa-leena.huttunen@oulu.fi

Table S1. Site-specific mean values for the variables used in regression model construction. BMI = bed movement intensity.

	BMI	Bryophyte cover (%)	Simpson's D	Connectivity[1]	Water temp.	Gamma
Ahvenoja	16.2	55.5	0.50	1500	11.7	58
Astumaoja	17.1	10.3	0.31	2350	12.7	54
Hangaspuro	19	0.6	0.65	2500	14.9	60
Isojärvenpuro	0	4.9	0.76	480	14.0	68
Juhtipuro	31.9	13.1	0.56	1000	10.9	50
Kantojoki	5.6	81.5	0.36	750	16.2	77
Kiutapuro	2.3	34.1	0.22	150	16.3	74
Kotioja	18.1	5.6	0.66	3300	11.6	56
Matinjärvenpuro	14.4	0.9	0.66	60	14.9	58
Merenoja	4.2	63.5	0.41	4500	15.8	66
Paahtojärvenjoki	17.6	40.1	0.44	1600	16.2	70
Pattasoja	0	65	0.23	900	11.5	55
Pessaripuro	4.2	19.5	0.35	350	15.2	54
Porontimajoki	8.8	86.6	0.50	900	14.1	74
Putaanoja	3.2	65	0.59	4200	11.5	60
Rysäjoki	8.8	5.5	0.53	1700	16.4	81
Rytipuro	1.4	3.2	0.40	200	16.5	66
Saaripuro	2.3	0	0.75	1100	16.3	69
Salmipuro	6.9	56	0.46	750	16.3	63
Sikalisko	15.3	50	0.43	700	11.9	50
Tulipuro	2.8	1	0.44	500	16.4	55
Uopajanpuro	20.4	51.2	0.64	3000	9.3	56
Vansselijoki	0.5	3.3	0.45	1000	16.2	71
Mean	9.6	31.1	0.5	1456	14.2	62.8
CV	0.9	0.9	0.3	0.9	0.2	0.1

[1]surface area (m^2) of riffles within a 1000 m buffer.

Fig. S1. Temporal variation (mean +1SD) in bed movement intensity.

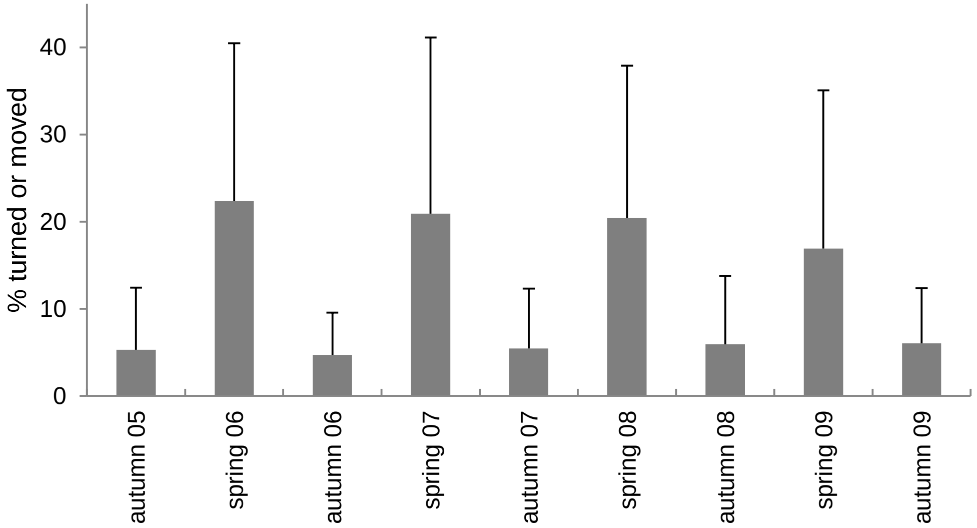

Supplement: Supplementary file 1 — Supplementary material [file 41598_2017_550_MOESM1_ESM.pdf]
